# A case of *bla*NDM-1-positive *Salmonella* Kottbus, Denmark, November 2020

**DOI:** 10.2807/1560-7917.ES.2021.26.26.2100569

**Published:** 2021-07-01

**Authors:** Hans Linde Nielsen, Philip K. Thomsen, Eva Litrup, Mia Torpdahl, Søren Overballe-Petersen, Frank Hansen, Anne Kathrine Schultz Christensen, Henrik Hasman

**Affiliations:** 1 Department of Clinical Microbiology, Aalborg University Hospital, Aalborg, Denmark; 2 Department of Clinical Medicine, Aalborg University, Aalborg, Denmark; 3 Department of Microbiology and Infection Control, Statens Serum Institut, Copenhagen, Denmark

## Abstract

We present a case of carbapenemase-producing *bla*NDM-1-positive *Salmonella* Kottbus in an 82-year-old Danish man. The *bla*NDM-1 was also identified in *Escherichia coli* and *Citrobacter freundii* in the same patient on the same 43 kb IncN2 plasmid, suggesting in vivo inter-species plasmid transfer. A NCBI BLAST analysis of the plasmid (pAMA003584_NDM-1) identified 12 highly similar plasmids, all originating from east and south-east Asia. This case could be the first confirmed case of *bla*NDM-1-positive *Salmonella* not related to travel outside Europe.

In Denmark, non-typhoidal *Salmonella* (NTS) is notifiable by the diagnosing laboratory and *S.* *enterica* subsp. *enterica* serovar Kottbus is a rare serovar, accounting for ca 1% of all NTS-cases registered over the past 20 years (https://statistik.ssi.dk). *S.* Kottbus has been isolated from poultry, cattle, pigs and reptiles [1] and has been identified in several outbreaks [2-5].

Carbapenems are not first-choice drugs for the treatment of *Salmonella*. However, the emergence of resistance to carbapenems, often last-line antimicrobial agents, is a major concern. In human *Salmonella* infections, five carbapenemases are of major clinical importance, namely *Klebsiella pneumoniae* carbapenemases (KPC; class A), New Delhi metallo-β-lactamase (NDM; class B), Verona integron-encoded metallo-β-lactamase (VIM; class B), and imipenemase (IMP; class B), and oxacillinases (OXA e.g. OXA-48; class D) [6]. 

We present a case of an NDM-1 carbapenemase-producing *S.* Kottbus, isolated in a Danish man who did not have travel history outside of Europe.

## Case report

The patient was an 82-year-old man with a recent diagnosis of inoperable lung cancer with no option for chemotherapy. In November 2020, he was hospitalised because of intermittent fever, abdominal pain, and diarrhoea for several weeks. On examination, he had tachycardia (112 beats/minute) and a body temperature of 37.4°C. Laboratory findings showed leucocytosis (34.1 × 10^9^/L; norm: 3.5–10.0) and elevated C-reactive protein (265 mg/L; norm: < 8.0). A computed tomography scan with contrast revealed bowel wall thickening in the left colon, suggestive of an underlying inflammatory or infectious condition. A stool sample taken on the day of admission was positive for *Clostridioides difficile* toxin B (Xpert *C. difficile* BT, Cepheid, Sunnyvale, California, United States (US)) and oral metronidazole treatment was initiated. In another stool sample also from the day of admission, *Salmonella* was isolated by routine methods and antimicrobial susceptibility testing was performed using the European Committee on Antimicrobial Susceptibility Testing (EUCAST) standardised disk diffusion method for *Enterobacterales* (Breakpoint table v10.0) [7]. Surprisingly, the isolate was resistant to meropenem (inhibition zone diameter: 11mm) and the NG-Test CARBA 5 (NG Biotech, Guipry, France) [8] was positive for NDM. The stool sample was also plated on chromID CARBA SMART agar (bioMérieux, Marcy l'Etoile, France), showing growth of *Escherichia coli* and *Citrobacter freundii*; both isolates were NDM-positive with the NG-Test CARBA 5. The diarrhoea symptoms subsided after a few days and the patient recovered. On day 8, he was discharged and treatment with oral metronidazole continued for a total of 10 days. After 2 weeks, he died at his home. 

A review of the patient's records clarified that, in September 2020, he had been at a resort on the Ionian Sea, Greece, for a week-long holiday. The patient and his partner stayed at an all-inclusive hotel; both had an onset of diarrhoea 2 days after arrival. The patient had many loose stools so he visited an outpatient clinic where he received intravenous rehydration and antibiotic therapy with oral cefuroxime. After the patient returned to Denmark, the diarrhoea symptoms subsided but the patient was hospitalised a few days later with kidney failure; a rectal swab taken as part of screening procedures for multidrug-resistant bacteria were negative. Throughout September and October, the patient was hospitalised for chronic kidney failure, and was eventually diagnosed with inoperable lung cancer. He was dialysed and received different antibiotic regimens, including oral metronidazole for recurrent diarrhoea. However, a stool sample for enteric pathogenic bacteria was not taken until the final hospital stay in November.

## Serotyping and genomic analysis at the National Reference Laboratory 

In 2018, the Danish Health Authority added carbapenemase-producing organisms (CPO) to the list of notifiable bacteria, and the Danish National Reference Laboratory (Statens Serum Institut (SSI)) carries out whole genome sequencing of all CPO isolates. At SSI, the isolate from the patient was serotyped as *S.* Kottbus based on the Kauffmann-White-Le Minor scheme, which was later verified using the sequence data to predict the serotype.

For short-read sequencing of the NDM-1-producing *S.* Kottbus, *E. coli* and *C. freundii*, DNA was extracted using DNeasy Blood and Tissue Kit (Qiagen, Hilden, Germany) and Nextera XT DNA Library Preparation Kit (Illumina, San Diego, California, US) was used before sequencing with a 2 × 151 bp paired-end Mid-Output kit (Illumina). The sequence reads are available from the European Nucleotide Archive (ENA; accession number: ERS6246687). For nanopore sequencing, DNA was extracted with the GenFind v3 (Beckman Coulter, Indianapolis, Indiana, US) using a DynaMag-2 magnet (Thermo Fisher Scientific, Waltham, Massachusetts, US). A library was prepared using the Rapid Barcoding Sequencing Kit (SQK-RBK004) and sequenced in a R10.3 flow cell (FLO-MIN111) with a MinION Mk1B (Oxford Nanopore Technologies, Oxford, United Kingdom (UK)). Using Guppy v4.2.2 (Oxford Nanopore Technologies), raw fast5 reads were base-called to fastq format in ‘high-accuracy’ configuration, demultiplexed and quality filtered to minimum q8. Then Illumina-Nanopore hybrid de novo genome assembly was run with Unicycler v0.4.8-beta [9].

**FIGURE 1:**
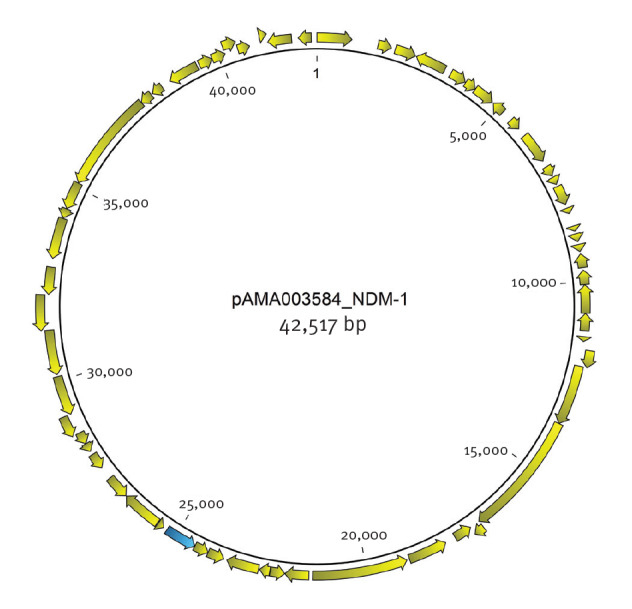
Map of the *bla*NDM-1-carrying 43 kb IncN2 plasmid pAMA003584_NDM-1 from an isolate of *Salmonella* Kottbus, Denmark, November 2020

The *E. coli* was identified as sequence type (ST)399 and *C. freundii* as ST18. The *bla*NDM-1 was identified in all three isolates placed on the same 43 kb IncN2 plasmid (pAMA003584_NDM-1; GenBank accession number: MZ004973), identified by PlasmidFinder v2.1 [10], as shown in Figure 1. 

GenBank accession number: MZ004973. The location of the *bla*NDM-1 gene is shown in blue.

A National Center for Biotechnology Information (NCBI) Basic Local Alignment Tool (BLAST) analysis of pAMA003584_NDM-1 identified 12 highly similar plasmids, all originating from east and south-east Asia (Table 1).

**Table 1:** A NCBI BLAST analysis of the *bla*NDM-1-carrying 43 kb IncN2 plasmid identified 12 highly similar plasmids all originating from east and south-east Asia

Plasmid	Organism	Accession length (bp)	GenBank accession number	Country of origin
pJN24NDM1	*Escherichia coli* strain JN24	41,190	MK368725.1	China
pC2972–5-NDM	*Klebsiella pneumoniae* strain C2972	51,995	CP039806.1	China
pCRE1.4	*Escherichia coli* strain CRE1	41,185	CP034398.1	Thailand
pNH25.5	*Klebsiella pneumoniae* strain NH25	38,383	CP024879.1	Thailand
pNDM-ECS01	*Escherichia coli* strain ECS01	41,190	KJ413946.1	Thailand
pC057_NDM1 DNA	*Klebsiella pneumoniae* C057	41,181	LC521837.1	Thailand
pC099_NDM1 DNA	*Klebsiella pneumoniae* C099	44,859	LC613145.1	Thailand
pCRE10.4	*Escherichia coli* strain CRE10	41,191	CP034403.1	Thailand
plasmid pTR3	*Klebsiella pneumoniae*	41,187	JQ349086.2	Singapore
pC2974–6-NDM	*Klebsiella pneumoniae* strain C2974	51,995	CP039800.1	China
pEcloNH77	*Enterobacter cloacae* strain NH77	41,179	CP040826.1	Thailand
plasmid pECL189–4	*Enterobacter hormaechei* strain 189	41,439	CP047969.1	China

The plasmid pAMA003584_NDM-1 has accession number MZ004973. Plasmids identified had 91–96% query coverage and 99.9–100% identity hits.

## Ethical Statement

A signed informed consent for publication from the deceased’s partner was obtained before submitting.

## Discussion 

In the European Union (EU)/European Economic Area (EEA), the notification rate of NTS was 20 cases per 100,000 inhabitants in 2019 [11]. In Denmark, the notification rate for NTS was almost identical (19.3 cases/100,000 inhabitants) in 2019, while the lowest rates were reported by Cyprus, Greece, Ireland, Italy, Portugal, and Romania (≤ 7.1 cases/100,000 inhabitants) [11].

Here we present the first confirmed case of *bla*NDM-1-positive *S.* Kottbus not related to travel outside Europe. The *bla*NDM-1 gene was also identified in *E. coli* and *C. freundii* on the same 43 kb IncN2 plasmid, suggesting an inter-species transfer of the *bla*NDM-1-carrying IncN2 plasmid in vivo.

The first NDM-producing NTS case, published in 2011, was a 60-year-old American man who was transferred from India to a hospital in the US where *bla*NDM-1-positive *Salmonella* Senftenberg was isolated from a perirectal surveillance culture [12]. Following this, other *bla*NDM-1-positive human NTS cases were described in connection to India, Pakistan and China, as reviewed by Fernández et al [6]. In 2015, Day et al. reported an isolate of *S.* Senftenberg from the UK, obtained in 2008 from faeces in an outpatient with unknown travel history. The isolate was resistant to ertapenem, but susceptible to meropenem and harboured the *bla*NDM-1 gene on a 53 kb IncX3 plasmid nearly identical (99.7%) to an IncX3-type *bla*NDM-1 plasmid from a *Raoultella planticola* detected in China [13]. During 2019, only a single carbapenem-resistant *Salmonella* Typhimurium var. O:5-negative-carrying *bla*OXA-48 was reported in the EU/EEA. In 2018, two isolates of *Salmonella* Kentucky (OXA-48-producing), and single isolates of *Salmonella* Corvallis (OXA-48-producing), *Salmonella* Rissen (KPC-producing) and *Salmonella* Typhimurium (VIM-producing) were identified, whereas no NDM-producing *Salmonella* were reported [14]. 

In the case presented here, we identified three bacteria in the same patient, all of which harboured the same carbapenem-resistance gene. Carbapenemase acquisition by an NTS from other *Enterobacteriaceae* in immunocompromised patients in a healthcare context has been suggested [6]. According to the patient’s partner, other guests at the resort also had diarrhoea; we can speculate that this patient may have eaten contaminated food at the resort in Greece, but the exact source of the *bla*NDM-1 plasmid remains unknown. Furthermore, we can only hypothesise which of the three bacteria first obtained the *bla*NDM-1 plasmid. Denmark has a low prevalence of carbapenemase-producing bacteria [15] and the fact that *S.* Kottbus is very rare in Denmark also suggests that the three bacteria had ‘spent time together’ in vivo. During the autumn, the patient was hospitalised and diagnosed with terminal lung cancer. He received different antibiotic regimens so, on the other hand, we cannot rule out an acquisition of the IncN2 plasmid by one of the three bacteria in Denmark.

## Conclusions

We describe the first case of *bla*NDM-1-positive *S*. Kottbus located on a 43 kb IncN2-plasmid from an 82-year-old man with terminal lung cancer detected in Denmark. The plasmid was also found in *E. coli* and *C. freundii* from the same patient*,* suggesting horizontal gene transfer. The patient had no known travel history outside Europe and could be the first confirmed case of *bla*NDM-1-positive *Salmonella* not related to travel outside Europe. Our finding underscores the importance of remaining vigilant for the potential risk of emerging resistance strains. In sum, we find any potential spread of NDM-1-producing NTS worrisome and emphasize the need for antimicrobial resistance surveillance in Europe, especially in countries where NDM-producing Enterobacteriaceae is spreading.

## References

[1] Toboldt A Tietze E Helmuth R Junker E Fruth A Malorny B. Molecular epidemiology of Salmonella enterica serovar Kottbus isolated in Germany from humans, food and animals. Vet Microbiol. 2014;170(1-2):97-108. 10.1016/j.vetmic.2014.01.020 24559660

[2] Centers for Disease Control and Prevention (CDC). Outbreak of Salmonella serotype Kottbus infections associated with eating alfalfa sprouts--Arizona, California, Colorado, and New Mexico, February-April 2001. MMWR Morb Mortal Wkly Rep. 2002;51(1):7-9. 11831433

[3] Enkelmann J von Laer A Simon S Fruth A Lachmann R Michaelis K Disentangling outbreaks using whole-genome sequencing: concurrent multistate outbreaks of *Salmonella* Kottbus in Germany, 2017. Epidemiol Infect. 2020;148:e51. 10.1017/S0950268820000394 32052718 PMC7078581

[4] Palmera-Suárez R García P García A Barrasa A Herrera D Investigation Team. Salmonella Kottbus outbreak in infants in Gran Canaria (Spain), caused by bottled water, August-November 2006. Euro Surveill. 2007;12(7):E070712.2. 10.2807/esw.12.28.03235-en 17868561

[5] Ryder RW Crosby-Ritchie A McDonough B Hall WJ 3rd. Human milk contaminated with Salmonella kottbus. A cause of nosocomial illness in infants. JAMA. 1977;238(14):1533-4. 10.1001/jama.1977.03280150103039 578222

[6] Fernández J Guerra B Rodicio MR. Resistance to carbapenems in non-typhoidal Salmonella enterica serovars from humans, animals and food. Vet Sci. 2018;5(2):40. 10.3390/vetsci5020040 29642473 PMC6024723

[7] The European Committee on Antimicrobial Susceptibility Testing (EUCAST). Clinical breakpoints and dosing of antibiotics. Växjö: EUCAST. [Accessed: 29 Nov 2020]. Available from: http://www.eucast.org/clinical_breakpoints/

[8] Jenkins S Ledeboer NA Westblade LF Burnham CA Faron ML Bergman Y Evaluation of NG-test carba 5 for rapid phenotypic detection and differentiation of five common carbapenemase families: results of a multicenter clinical evaluation. J Clin Microbiol. 2020;58(7):58. 10.1128/JCM.00344-20 32376668 PMC7315033

[9] Wick RR Judd LM Gorrie CL Holt KE. Unicycler: Resolving bacterial genome assemblies from short and long sequencing reads. PLOS Comput Biol. 2017;13(6):e1005595. 10.1371/journal.pcbi.1005595 28594827 PMC5481147

[10] Carattoli A Zankari E García-Fernández A Voldby Larsen M Lund O Villa L In silico detection and typing of plasmids using PlasmidFinder and plasmid multilocus sequence typing. Antimicrob Agents Chemother. 2014;58(7):3895-903. 10.1128/AAC.02412-14 24777092 PMC4068535

[11] European Food Safety Authority; European Centre for Disease Prevention and Control. The European Union One Health 2019 Zoonoses Report. EFSA J. 2021;19(2):e06406. 10.2903/j.efsa.2021.6406 33680134 PMC7913300

[12] Savard P Gopinath R Zhu W Kitchel B Rasheed JK Tekle T First NDM-positive Salmonella sp. strain identified in the United States. Antimicrob Agents Chemother. 2011;55(12):5957-8. 10.1128/AAC.05719-11 21968356 PMC3232776

[13] Day MR Meunier D Doumith M de Pinna E Woodford N Hopkins KL. Carbapenemase-producing Salmonella enterica isolates in the UK. J Antimicrob Chemother. 2015;70(7):2165-7. 10.1093/jac/dkv075 25795771

[14] European Food Safety Authority; European Centre for Disease Prevention and Control. The European Union Summary Report on Antimicrobial Resistance in zoonotic and indicator bacteria from humans, animals and food in 2018/2019. EFSA J. 2021;19(4):e06490. 10.2903/j.efsa.2021.6490 33868492 PMC8040295

[15] DANMAP. Use of antimicrobial agents and occurrence of antimicrobial resistance in bacteria from food animals, food and humans in Denmark. ISSN 1600-2032. Copenhagen: Statens Serum Institut, National Food Institute, Technical University of Denmark; 2018. Available from: www.danmap.org

